# Effects of mobile health education on sexual and reproductive health information among female school-going adolescents of rural Thailand

**DOI:** 10.12688/f1000research.53007.1

**Published:** 2021-06-08

**Authors:** Premyuda Narkarat, Surasak Taneepanichskul, Ramesh Kumar, Ratana Somrongthong

**Affiliations:** 1 Chullalongkorn University, College of Public Health Sciences, Bangkok, Thailand; 2 Public Health, Health Services Academy, Islamabad, 44000, Pakistan

**Keywords:** Sexual Health, Reproductive Health Literacy, teenage pregnancy, sex education, mobile health, effective health education.

## Abstract

**Background**: Adolescent pregnancy is one of the major public health issues globally, as well as in Thailand. Sexual health literacy (SHL) has been a proved effective intervention for preventing teenage pregnancy.

The objective of this study was to evaluate the effects of mobile messages to improve sexual and reproductive health literacy among secondary school female students.

**Methods**: A comparative cross-sectional study with pre–post design was conducted in two secondary schools of rural Thailand. 128 respondents were selected through a simple random sampling method; equal number of female secondary school students were selected from each school. Health education through mobile messages on sexual and reproductive health literacy were delivered in one group while the other group was observed through routine care for 24 weeks. Baseline (pre) and endline (post) measurement was taken to compare the effects of mobile messages. Study was ethically approved by the institutional review board of Chulalongkorn University, Thailand.

**Results**: Both groups were same at baseline and found statistically non-significant (p>0.05). After the 24-week health education program, the mean scores of health education groups was found statistically significant (p<0.05), while the mean score in observed group did not show any statistical change (p>0.05) . Level of sexual health literacy scores among female students in the health education group was found statistically significant (p<0.05) in all four domains. While, the observation group was remained same at both measurements pre and post (p=0.521).

**Conclusion**: The study concluded that the mobile messages have proved an effective information method for sexual and reproductive health information among female secondary students of rural Thailand.

## Introduction

Adolescent pregnancy is a well-known reproductive health problem in the teenage population of Thailand. Moreover, this age group is more prone to pregnancy due to their active sexual and biological health. ^1^ Studies have found that 41% of adolescents were sexually active, and about 20% were engaged in risky sexual behaviors. ^1, 2^ The reported data showed that, annually, about 47 childbirths occurs in 1,000 adolescent mothers in the world. ^3^ The United Nations Children's Fund data from Thailand, 2016–2017, found that the prevalence of adolescent pregnancy in the age group of 15–19 years was 51%. ^4^ Moreover, other sources also revealed that the birth rates among adolescents aged 15–19 years during in 2013, 2014 and 2015 was 51.1, 47.9 and 44.8 per 1,000 adolescents women, respectively. ^5, 6^ In 2018, the childbearing rate among adolescents aged 10–19 years increased to 12.9%, which is an alarming signal for policy makers in sexual and reproductive health in the country. ^7^ Hence, the situation of adolescent pregnancy has become a predominant problem in Thailand that needs urgent interventions. ^8^ Moreover, it has been observed through the evidence that the this number of adolescent pregnancy is more in the southern part of Thailand including Chumpon province, Krabi province, and Nakhon Si Thammarat province. ^9^ This problem of adolescent pregnancy has not yet been resolved. Moreover, proper information and knowledge for adolescents on sexual health literacy, early aged pregnancy, sexually transmitted diseases (STD), childbearing information and care are highly needed. ^10^

Sexual health literacy is very effective approach for improving an individual's cognitive and social skills that result in developing a positive behavior. ^11^ Sexual health literacy implies the achievement of knowledge levels, personal skills, and confidence to take action for personal and community health improvement by changing personal lifestyles and living conditions. Improving adolescents' access for sexual and reproductive health information, and their capacity to use it effectively, sexual health literacy should be provided and tested among the younger population of Thailand. Therefore, this study was conducted to evaluate the effects of mobile messages to improve sexual health literacy among secondary School female students of rural Thailand.

## Methods

### Study design

A comparative cross-sectional study with pre–post design was conducted at two secondary Schools of Nakhon Si Thammarat and Krabi provinces of southern Thailand from February–July 2020. Secondary school female students were the respondents in this study. One school was randomly selected as a group with health education and other school as the observation group.

### Sample size and selection

The sample size was calculated using 80% power, 0.05 alpha with 10% difference, and 128 students were selected for this study. An equal number of 64 female students were randomly selected from each school and allocated for both groups.

### Data collection

Data collectors were trained on how use the data collection tool, and briefed on the study prior to starting the study. The survey technique was face-to-face interviews. The process took approximately 30 minutes for each participant. The internal consistency of the questionnaires was measured through Cronbach alpha (0.9) was used to collect sociodemographic information and sexual health literacy as per the objectives. ^12^ Health education group was given; 24-week program consisted of a weekly sexual health education animation on Facebook and a LINE application message on the importance of sexual health was given. The eight-week sexual health education topics were delivered once a week for 1.5 minutes through the LINE application. All the selected intervention participants gave responses *via* Facebook twice a week for up to 24 weeks. Female secondary school students received three LINE messages each week, sent in the evening hours. They continued receiving such messages from recruitment (at the first week of the section education animation on Facebook period intervention) to 24 weeks at the end of the intervention. A total of 72 LINE messages were developed and three line messages were sent per week. Furthermore, the activities included the presented infographic which the participants received for each topic of animation. The contents were the same as the knowledge on Facebook's contents. The control group received only the routine education. Weekly messages were sent for four weeks' time on situation of unintended pregnancy and contraception for accessing, understanding the actual situation, appraising the contexts of this problem and finally applying this problem in real life. A complete information with different pictures and real-life examples on sexual health were also shared with the respondents. Participants were briefed that they should contact a free help line to get more information in this regard.

### Statistical analysis

Data were analyzed using the Statistical Package for Social Science **(IBM SPSS Statistics, RRID:SCR_019096)** Software version 22 licensed for Chulalongkorn University; JASP ( **RRID:SCR_015823**) is an open access alternative to SPSS. The sexual health literacy (SHL) score was calculated by using the formula “Index - score = (mean–minimal value of mean)×(50/3)”, scaled by four levels: inadequate: 0–25; problematic: >25–33; sufficient: >33–42; and excellent: >42–50. Both the formula and the scales were adopted from the European Health Literacy Survey (HLS-EU-Q47) method. ^13^ The homogeneity of baseline characteristics was analyzed by using Chi-squared test for nominal data. In inferential statistics, one-way ANOVA repeated measurement was used for comparison of mean changes for all time points within intervention and control groups. The pairwise comparison of one-way ANOVA repeated measurement was used for intervention and control groups at different times of measurement. The independent t-test was used to compare the change between intervention group and control group for all time points. The level of significance was set at a p-value < 0.05 for all statistical analyses.

### Ethics and consent

This study was approved by the Research Ethics Review Committee for Research Involving Human Research Participants, Health Sciences Group, Chulalongkorn University (COA No. 252.2/62). Data was collected from the respondents after taking the written informed consent from their parents. Administration approval was taken from the head of both schools.

## Results

Sociodemographic characteristics of the respondents of both the health education and observation groups are explained in Table 1. The comparison of general characteristics between the two groups were not different in terms of grade point average, age, allowance, and parent's marital status. Most of the female students were 14-year-old students of grade 8, and obtained grade point average over 3.0. The characteristics between two groups were not statistically different in term of grade point average (p = 0.439), age (p = 0.312), monthly income (p = 0.220), living condition (p = 0.410), and their parent's marital status (p = 0.465). Hence, both groups have similar socio-demographic information selected for the study.

**Table 1.** Baseline characteristics of participants af both groups (n = 128).

Variable		Health education (n = 64)	Observation (n = 64)	p-value
Grade	Current grade point average GPA ^a^	3.20 ± .54	3.28 ± .58	0.439 ^b^
Age	Students	14	14	0.312
Income	Average Income ^a^	3064.06 ± 981.96	3349.69 ± 1571.59	0.220 ^b^
Living condition	Parents	58 (90.6%)	55 (85.9%)	0.410 ^c^
	Relatives	6 (9.4%)	9 (14.1%)	
Marital status of parents	Married	61 (95.3%)	59 (92.2%)	0.465 ^c^
	Separated	3 (4.7%)	5 (7.8%)	

^a^ Value are mean ± SD.

^b^ P-value resulted from the Independent t-test.

^c^ P-value resulted from the Chi-square-test.

The level of sexual health literacy scores calculated as per index score mentioned in the method section among secondary school female students of both groups shows statistical significant difference in mean, SD and range (p = 0.000) after the health education. Group with health education was observed with significant changes with domains like; sufficient (76%), problematic (22%) and inadequate only (2%). However, the observation group remained the same without any change. The health education group reported only 1 (2%) participant with inadequate but on other hand, observation group showed all their participants with inadequate sexual health literacy group ( Table 2).

**Table 2.** Level of sexual health literacy scores among secondary school female students of health education and observation groups.

Level of sexual health literacy scores	Health education group n(%)	Obseravtion group n(%)	p-value
Mean, SD (Min-Max)	$\bar{x}$ = 34.44, SD 3.21, (23.50-39.74)	$\bar{x}$ = 9.98, SD 2.02, (6.84-17.52)	
Inadequate (0-25)	1 (2)	64 (100)	0.000 *
Problematic ( >25-33)	14 (22)	-	
Sufficient (>33-42)	49 (76)	-	
Excellent (>42-50)	-	-	

* Significant at 0.05 level.

Both groups were compared through average mean score measured by calculation of all four domains like: accessing, understanding, appraising and applying. The observation group remained the same at both measurements: pre and post (p = 0.521). However, the health education group showed statistically significant differences in all four domains (p = 0.000). This shows that the health education group has received positive information as compared to observation group that remained same ( Table 3).

**Table 3.** Comparision of sexual health literacy scores among secondary school female students of both group (Pre vs Post of the health education).

Sexual Health Literacy	Health education (mean SD)			Observation (mean SD)		
	Pre	Post	p-value	Pre	Post	p-value
Accessing	1.760 (0.206)	3.220 (0.242)	0.000	1.748 (0.128)	1.762 (0.188)	0.521
Understanding	1.495 (0.145)	2.992 (0.211)	0.000	1.473 (0.131)	1.620 (0.210)	0.411
Appraising	1.525 (0.200)	3.067 (0.305)	0.000	1.565 (0.192)	1.614 (0.184)	0.103
Applying	1.313 (0.140)	3.017 (0.312)	0.000	1.325 (0.106)	1.462 (0.179)	0.351

**Figure 1.**
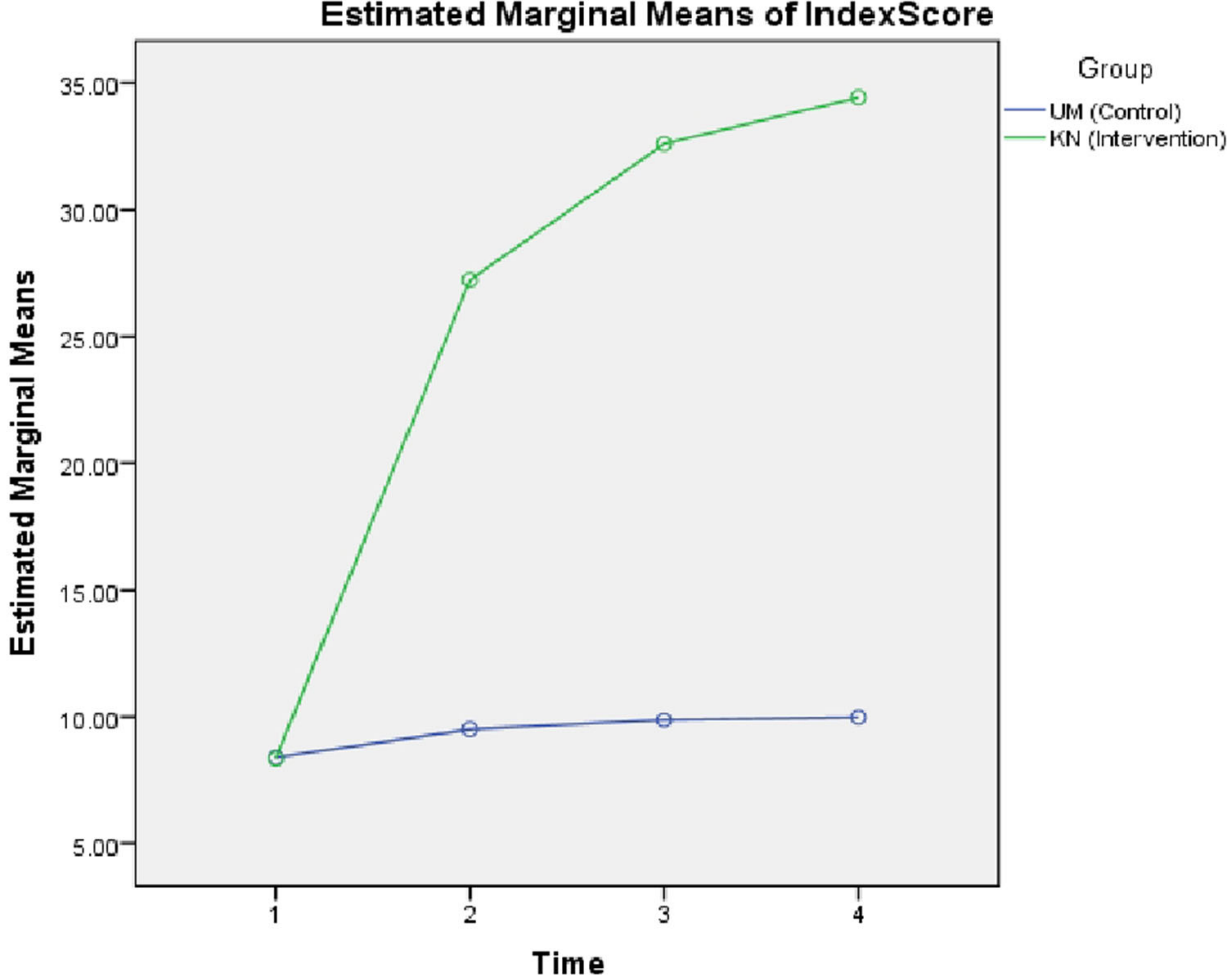
Estimated marginal means of index score.

The magnitude of change in the mean score of sexual health literacy scores was significantly higher in the health education group than the observation group ( Figure 1).

## Discussion

Students with good educational record perceived health education messages in better way as compared to those who had poor academics performance. This is consistent with a study showed that adolescents who attended school in urban setting had significantly higher score on sexual health literacy and knowledge. ^12, 13^ Female adolescents who are in the middle of psychosocial development phases, they undergo various changes in terms of their physical, cognitive, emotional, and sexual development at this stage. ^14^ These changes can lead to major risk factors for premarital sexual behaviour, adolescent pregnancy, abortion, and sexually transmitted infections. Health literacy is also effected by factors such as education, ethnicity, gender, age, profession, income, culture, language, and social support. ^15, 16^ However, this study focused on several variables including age, grade point average, average allowance, parent's marital status, and living with parents that also indirectly affected the sexual and reproductive health of the respondents. This can be assumed that students who are performing well in school may have higher knowledge and understanding toward sexual health literacy.

It was found that the measurement of the sexual health literacy scores (index) of the health education group in the baseline and endline had increased respectively through duration of the health education. There were statistically significant changes of the overall index scores before and after the health education program of the health education group. These findings are consistent with a study conducted on sex education program and prevention among grade 8 female students, where it was found that the experimental group had significantly higher levels of knowledge about preventing sexually transmitted disease and unwanted pregnancy, attitudes towards sexual behavior, self-efficacy in prevention of undesirable sexual behaviors, self-esteem, and sexual behaviors. This can be explained by the score of female students who received program information that was relevant to sexual health literacy which is consistent with accessing, understanding, appraising, and applying. Comparison of sexual health literacy scores (index) of the health education and observation groups at different time of measurement showed that the health education group's sexual health literacy scores had changed as compared to baseline. However, the observation group's sexual health literacy scores did not change and remained same in both pre and post measurements. This can be explained by that the observation group did not receive the program; moreover, people can improve their literacy skills through intensive health education. The mean scores in the comparison group gradually declined in all four domains. The increasing trend in scores from the baseline to endline can be explained by the health education activities that comprised all four domains of sexual health literacy. Moreover, the health education group received health education through social media model plus line messages. In addition, the program designed was based on information, motivation and behavioral skills health education that is relevant to sexual health literacy and cover assessing, understanding, appraising and applying in display of info-graphic and animations. Participants randomly received rewards when joining the activities through LINE. This is similar to the health education study which found that the mean scores of health literacy were higher than those in the observation group after receiving the Sex Must Safe Program, the health education program, which included all the elements of health literacy. ^16^

## Conclusion

This study concluded that the mobile messages are proved to be effective among adolescents to increase their sexual and reproductive health literacy and this could be replicated in similar settings of the country to promote sexual and reproductive health and control the teenage pregnancy issues in the country.

## Limitations and recommendations

The sample size of this study was small and population in the study was secondary school female students only. The areas of the study were purposively selected; hence the results of the study may not be generalizable to other locations and population. This Social Media Model should be recommended to policy makers in order to be applied in the prevailing situation. Implementation and modification of the Social Media Model's contents should be performed by relevant government authorities in order to be applicable for general population; especially for a rapid observation and prevention of teenage pregnancy. Limited time of measurements in the intervention group could have an effect on the knowledge of the participants in medium or long term knowledge, hence this study focuses on a short-term evaluation.

## Data availability

### Underlying data

Open Science Framework: Underlying data for ‘Effects of mobile health education on sexual and reproductive health information among female school-going adolescents of rural Thailand’. http://www.doi.org/10.17605/OSF.IO/WPUCZ

Data are available under the terms of the Creative Commons Zero “No rights reserved” data waiver (CC0 1.0 Public domain dedication).

### Author's contributions

PN conceived the study design and ST supervised the data collection. RK, did the data analyses and drafted the paper. RS conducted the critical review and added the intellectual content to the paper. All authors read and approved the final draft.
