# Supplementary material for: Effects of mobile health education on sexual and reproductive health information among female school-going adolescents of rural Thailand
Source: F1000Res. 2021 Jun 8;10:452. [Version 1] doi: 10.12688/f1000research.53007.1 (PMC8276189; doi:10.12688/f1000research.53007.1)
Supplement: Peer review history

# Peer review: Reviewer response for version 1

**DOI:** 10.5256/f1000research.56348.r87017

**Published:** 2021-07-09

**Authors:** Abdul Wali Khan

**Affiliations:** 1 Ministry of National Health Services, Regulations & Coordinations, Islamabad, Pakistan

The authors have conducted study on a very important public health area. I reviewed the article and have made the following observations as part of my review:

1. In the title, please mention the place of study in Rural Thailand.
2. In the Abstract also mention the place of study in both the objectives and methodology sections. 
3. Some more literature especially about explanation of adolescence...as risk group; and also on effects of sexual literacy on adolescence and as well as on pregnancy's in this group need to be added.
4. In the Methods section, study design i.e. pre-post design is not clear. It should be further elaborated. It is stated that school was randomly selected...it should be explained...how this was selected from how many schools. It is not clear how the study participants were selected inside the school. Please explain it.
5. In the Results section, in the description also mention the exact number with percentages. Also explain the captions in Figure 1 as well as the tables. If you have bar-charts, could be incorporated in the Result Section.
6. In the Discussion section, individual findings of the study need to compared with the similar findings of the relevant studies (especially 2nd Paragraph of the Discussion).

The above changes need to be made before indexing. The authors have conducted study on a very important public health area.

Is the work clearly and accurately presented and does it cite the current literature?

Yes

If applicable, is the statistical analysis and its interpretation appropriate?

Yes

Are all the source data underlying the results available to ensure full reproducibility?

Partly

Is the study design appropriate and is the work technically sound?

Partly

Are the conclusions drawn adequately supported by the results?

Yes

Are sufficient details of methods and analysis provided to allow replication by others?

Partly

Reviewer Expertise:

MSPH, PhD (Public Health)

I confirm that I have read this submission and believe that I have an appropriate level of expertise to confirm that it is of an acceptable scientific standard.

# Peer review: Reviewer response for version 1

**DOI:** 10.5256/f1000research.56348.r87018

**Published:** 2021-06-15

**Authors:** Abdelazeem Mohammed

**Affiliations:** 1 Department of Bacteriology, Immunology, and Mycology, Faculty of Veterinary Medicine, Suez Canal University, Ismailia, Egypt

This is an excellent academic research study  aimed to evaluate the effects of mobile messages to improve sexual and reproductive health literacy among secondary school female students in Thailand. The aims, objectives and study design including sample size and data collection methods are appropriate and sufficiently explained by the authors. The authors have cited the local and contextual literature and well justified their findings in discussion part. Ethical considerations, a written consent was taken from the parents that is an important element of this study. However, considering the importance of the topic under study, I would suggest the investigators need to provide more details on the recruitment of the respondents.  Additionally, the manuscript should be revised for English editing and grammar mistakes.

**Comments:**

- Title: I think the work would benefit from the title that contains the main conclusion of the study (should be derived from the conclusion), please modify the title.
- Abstract: Please,  highlight the main conclusion of the study. 
- Methods: Please, add more details about the study design with specific references.
- Results: Good presentation.
- Discussion: The authors are advised to illustrate the real impact of their findings without the repetition of results. 
- Conclusion: Should be rephrased to be sound better. A real conclusion should focus on the question or claim you articulated in your study, whose resolution has been the main objective of your paper? That question now needs to be re-invoked and definitively answered. More still, you need to leave your reader with a higher level of insight into your topic.

Is the work clearly and accurately presented and does it cite the current literature?

Yes

If applicable, is the statistical analysis and its interpretation appropriate?

Yes

Are all the source data underlying the results available to ensure full reproducibility?

Yes

Is the study design appropriate and is the work technically sound?

Yes

Are the conclusions drawn adequately supported by the results?

Yes

Are sufficient details of methods and analysis provided to allow replication by others?

Yes

Reviewer Expertise:

NA

I confirm that I have read this submission and believe that I have an appropriate level of expertise to confirm that it is of an acceptable scientific standard.
